# Healthcare Professionals’ Attitudes and Expectations Toward Digital Inpatient-like Psychotherapy: A Qualitative Interview Study

**DOI:** 10.3390/healthcare14183045

**Published:** 2026-09-16

**Authors:** Patrick Jonas Wollenberg, Tania Lalgi, Lucy Ann Gresser, Christoph Jansen, Rebekka Robitzsch, Alexander Diel, Martin Teufel, Alexander Bäuerle, Anita Robitzsch

**Affiliations:** 1 Clinic for Psychosomatic Medicine and Psychotherapy, University of Duisburg-Essen, LVR-University Hospital Essen, 45147 Essen, Germanyanita.robitzsch@lvr.de (A.R.); 2 Center for Translational Neuro- and Behavioral Sciences (C-TNBS), University of Duisburg-Essen, 45147 Essen, Germany

**Keywords:** mental healthcare, e-mental health, eHealth, digital therapy, digital intervention

## Abstract

**Background:** Digitalization in medicine is becoming increasingly important to ensure low-threshold, comprehensive, flexible, multimodal, and multiprofessional treatment. This qualitative interview study explores the nuanced perspectives of mental health professionals regarding a digital inpatient-like psychotherapy concept. **Methods**: This study employed semi-structured qualitative interviews to assess attitudes and expectations toward a digital inpatient-like psychotherapy concept among mental health professionals in Germany. The resulting data were analyzed using a hybrid deductive–inductive codebook thematic analysis involving a consensus-based coding process by three independent researchers. **Results:** A total of *n* = 25 interviews with physicians (assistant physicians, specialists), psychotherapists, nurses, psychologists, and individuals from other professions were included in the data analysis. The interviewees held various roles and brought diverse levels of experience to the study, in the context of inpatient mental healthcare. The general attitude toward digital inpatient-like psychotherapy was positive, particularly when viewed as a supplement to traditional inpatient mental healthcare and implemented in a hybrid model. However, the technical infrastructure and the suitability of patient cohorts warrant further examination. **Conclusions**: Digital inpatient-like psychotherapy offers a promising approach to enhancing mental health treatment. Mental health professionals generally expressed a positive attitude toward such concepts, though certain structural and contextual conditions—such as establishing adequate technical support structures and improving the digital literacy of mental health professionals—must be addressed prior to implementation.

## 1. Introduction

According to the World Health Organization (WHO), mental health constitutes “an integral part of our general health and well-being and a basic human right” [1]. Despite this recognition, mental health disorders are increasingly prevalent worldwide and rank among the most common illnesses [2,3]. Addressing the escalating demand for mental healthcare necessitates the allocation of substantial resources and time, a factor that contributes to long waiting times [4]. In Germany, deficiencies in psychotherapeutic service provision, e.g., long waiting times, have been evident since the introduction of demand-oriented planning [5]. Despite the implementation of various reforms, treatment delays and limited access to psychotherapy persist [6,7].

The mental health system is experiencing significant challenges, including prolonged waiting times and shortages in staff, which are further compounded by the rise in case numbers in both outpatient and inpatient care settings. Although inpatient treatment remains essential for patients requiring intensive, multimodal, and multiprofessional care, the average length of stay has decreased [8,9]. Despite the augmentation of capacity to address escalating demand, this phenomenon has concomitantly engendered an elevated risk of rehospitalization and the emergence of so-called “revolving-door” patients [10]. Consequently, alternative treatment models, including inpatient-equivalent psychiatric treatment (StäB), outpatient acute psychiatric treatment at home (APAH), and outpatient intensive complex treatment (AMBI), have gained increasing attention [11]. These approaches have also been applied in the domain of gerontopsychiatry [12] and in StäB with elderly people [13]. Rauschenberg et al. [11] concluded that digital treatment models have the potential to improve treatment effectiveness and cost-efficiency. However, the necessity of additional research regarding the quality and safety parameters of these models was also stressed [11]. A 12-month follow-up study revealed that StäB demonstrates superiority over inpatient treatment, as evidenced by its reduced readmission rates to the hospital [14].

Nevertheless, a persistent shortage of mental health professionals (MHPs) in nursing and psychotherapy further exacerbates existing limitations [15]. The aforementioned structural challenges are particularly evident in inpatient settings. The time-intensive nature of care, in conjunction with the shortage of qualified personnel, poses a significant burden for both patients and practitioners.

Germany‘s healthcare system is facing increasing nationwide financial pressure, affecting the provision of hospital care [16]. This imbalance has led to the closure of smaller, non-specialized hospitals due to inadequate refinancing [17]. Furthermore, constrained financial resources have impeded the rate of digital transformation in the healthcare sector, resulting in its lagging position in comparison to other industries. This situation underscores the pressing need for the implementation of digital transformation strategies. In Germany, psychosomatic medicine is regarded as a distinct medical specialty, distinct from and independent of psychiatry [9].

To address the aforementioned issues, it is recommended that the implementation of digitalization be considered. This process has gained increasing attention in the healthcare field due to its potential benefits. For instance, a study reported that 28.5% of healthcare professionals recommended e-mental health interventions [18]. Among these professionals, 70.2% of specialized physicians, 70.8% of psychotherapists, and 60% of other clinical staff reported using these interventions for self-help, therapeutic assignments, and aftercare [18]. Digital tools, including video consultations, digital diagnostics, home monitoring [19,20], digital health applications (DiGA) [21], and virtual reality (VR) interventions [22,23], may offer additional opportunities for both patients and providers. Video consultations have gained broad acceptance among providers, with 88.5% of providers expressing willingness to continue using them [19]. Recent developments, including electronic prescriptions (e-Rezept), electronic health records (ePA), and DiGA, demonstrate ongoing efforts to advance and modernize digital healthcare delivery in Germany [24,25,26]. Digitalization has already become an integral part of the medical field in several countries, whereas in Germany, the transition to digitalization is proceeding more slowly [27].

Digitalization may offer several advantages, including reduced waiting times, decreased stigmatization [10], improved accessibility and flexibility, e.g., for individuals in rural areas or those working full time [28,29,30]. The availability of care and the concomitant reduction in workload and costs for healthcare staff are other potential advantages [16,31]. However, existing research has mainly focused on the efficacy of specific digital interventions [32], such as web-based programs [28], or online cognitive behavioral therapy (iCBT) [33,34,35]. However, little is known about MHPs’ perspectives on comprehensive digital therapy models, particularly those that mimic the structure of inpatient care.

To address this gap, the theoretical concept of digital inpatient-like psychotherapy (DIPT) has been proposed. The DIPT initiative aims to digitally replicate fundamental components of inpatient treatment through an integrated approach, integrating therapeutic, educational, monitoring, and interprofessional components. The DIPT model is designed to function as an integrated, structured care system of inpatient treatment. The term “inpatient-like” is not merely a reference to the delivery of psychotherapy via digital means. Rather, it signifies the transposition of the fundamental structural elements that define inpatient treatment into a digital environment. It has been demonstrated that this system has the capacity to promote interprofessional communication, optimize workflows, reduce administrative burden, and enable different levels of treatment intensity. The objective of this initiative is to promote patient engagement and enhance the focus on the therapeutic process. Conversely, conventional telepsychotherapy or blended care may primarily use digital communication or digital components to supplement conventional outpatient treatment without reproducing this level of structure, intensity, and multiprofessional coordination.

According to the Unified Theory of Acceptance and Use of Technology (UTAUT), technology adoption may be influenced by performance expectancy, effort expectancy, social influence, and facilitating conditions [36]. These dimensions provide a useful framework for interpreting factors that may shape MHPs’ acceptance of DIPT.

Despite the apparently high level of acceptance of digital interventions, their integration into routine clinical care remains a significant challenge [18]. Successful technology integration requires the involvement of relevant professional groups, as MHPs‘ attitudes and perspectives can influence technology acceptance and adoption. Therefore, interdisciplinary and multiprofessional collaboration is important to ensure that the perspectives and needs of different professional groups are considered during implementation [34,37].

This qualitative interview study sought to investigate MHPs’ attitudes and expectations regarding the theoretical DIPT concept. Through semi-structured interviews, this study aimed to identify critical prerequisites and barriers to the successful integration of DIPT into routine clinical practice. Assessing MHPs’ attitudes toward digital psychosomatic medicine is essential to understand their openness to this form of technology and the preferred implementation in this field. This theoretical concept has previously been explored in two publications from the perspectives of patients and students [38,39].

## 2. Materials and Methods

### 2.1. Study Design and Quality Control

The present study employed a qualitative study design, with semi-structured interviews conducted at a single assessment point. Ethical approval was obtained from the Ethics Committee of the Medical Faculty of the University of Duisburg-Essen (23-11642-BO).

In order to guarantee the rigor and validity of the research, the study was reported in accordance with the Consolidated Criteria for Reporting Qualitative Research (COREQ) guidelines, which were developed by Tong et al. (2007) [40], and followed the established qualitative research standards outlined by Yardley (2000) [41]. The COREQ checklist is included in Appendix A.2. In order to enhance the analytical rigor of the study, the initial coding of the data was completed by two independent researchers (TL and PW). Furthermore, a third independent researcher performed cross-coding. Adopting Kuckartz’s (2022) [42] methodology for structural guidance, this process successfully addressed initial discrepancies and achieved a consensus-based coding agreement on the overarching themes, themes and subthemes. As part of an iterative process, the researchers met regularly to discuss inconsistencies, align their interpretations, and jointly develop an understanding of the data based on the transcript analyses [43]. Throughout the study, the research team held regular online and in-person meetings to ensure consistency and quality throughout the analytical process.

### 2.2. Assessment, Semi-Structured Interview

The interview guide, originally developed in German, consisted of questions aligned with the study’s objectives. Prior to the commencement of the recruitment process, pilot interviews were conducted with the objective of refining the interview guide and estimating the expected duration of the interviews.

At the beginning of each interview, participants completed a brief questionnaire assessing sociodemographic data such as age, gender, professional role within the hospital, leadership position, and duration of employment. Additionally, participants were asked six questions regarding their use of digital media in the workplace. The final sections assessed participants’ familiarity with the concept of DIPT. Participants familiar with the concept were asked to define it in their own words. A standardized explanation was then presented, followed by an opportunity for participants to seek clarification. The full interview guide is provided in Appendix A.1.

The interview guide was composed of two primary sections: (1) Attitudes toward digitalization and DIPT (eight open-ended questions), and (2) Expectations toward DIPT (nine open-ended questions). The interview was concluded with two final questions: one asked participants to discuss any issues not yet mentioned, and the other asked the participants to highlight the three most important aspects mentioned during the interview. Open-ended questions were chosen to elicit rich, in-depth responses while allowing participants to reflect freely on their experiences in a personalized manner.

### 2.3. Recruitment and Participants

A total of 25 interviews were conducted between 27 March and 17 July 2024, including seven physicians (four assistant physicians and three specialists), six psychotherapists, five nurses, three psychologists, and four individuals from other professions (one experienced involvement specialist, one nutritionist, one social worker, and one special therapist). The participants were recruited from the Clinic for Psychosomatic Medicine and Psychotherapy at the LVR-University Hospital Essen. The voluntary nature of participants in the study may have resulted in a self-selection bias, as individuals with particular interest in the topic may have been more likely to participate. In addition, courtesy bias cannot be ruled out, as participants may have been inclined to provide more favorable responses. MHPs were informed about the study via email and provided with an informed consent form prior to the interviews. The participants were provided with the study requirements and general study information in both oral and written formats. Thus owing, written informed consent was obtained from each participant. Recruitment was also conducted using flyers posted on relevant wards to achieve universal coverage of all MHPs. The hospital was selected because of its large psychosomatic department and its established research focus in psychosomatic medicine. In Germany, psychosomatic medicine is a distinct medical specialty, separate from psychiatry. The professional groups were selected based on their direct involvement in patient care and therapy.

The recruitment process was conducted from 27 March to 18 June 2024, with the final interview conducted on 17 July 2024. Of the 41 MHPs who were contacted, twenty-five consented to participate, resulting in a response rate of 60.98%. A total of sixteen MHPs opted to decline or did not respond to the invitation to participate. The reasons for non-participation were unknown. Inclusion criteria required MHPs to have experience in psychotherapeutic treatment and active involvement in a psychotherapeutic setting. This included both MHPs in training and those with advanced training. All participants were required to demonstrate sufficient proficiency in the German language. The exclusion criteria encompassed two key factors. First, individuals under the legal age of 18 were ineligible to participate. Secondly, individuals with a familial relationship with PW were excluded, as PW conducted all interviews and interacted directly with the participants. PW received methodological guidance and supervision from an experienced researcher. Furthermore, participants were required to be employed at the Clinic for Psychosomatic Medicine and Psychotherapy at the LVR-University Hospital Essen.

### 2.4. Data Collection

The data collection process was terminated upon reaching thematic saturation [44], defined as the point at which no new themes or properties emerged from the data [45]. Saturation was assessed across the entire dataset, rather than separately within the various MHP groups. Throughout the data collection and analysis process, TL, SB, and PW convened regular meetings to review the emerging codes, themes, and properties, and to assess whether additional interviews contributed to new aspects to the development of thematic structure and monitor saturation. The final interviews did not yield any themes or subthemes at the level of the overall dataset.

Thematic saturation was assessed at regular intervals during data collection, including after every fifth interview. Saturation was assessed at the level of the overall dataset rather than separately within professional groups. The analysis of the final three interviews revealed that no novel codes, themes, or subthemes emerged, indicating that saturation had been reached.

A total of twenty-four interviews were conducted in person, while one interview was conducted online. All interviews were conducted in German by PW (doctoral student), who had no prior relationship to the interviewees, and participation was voluntary. PW was supported by an experienced researcher, who provided methodological guidance and consultation throughout the interview process, while PW independently conducted the interviews and interacted directly with the participants. The semi-structured interviews were audio-recorded using a password-protected device and transcribed using f4X transcription software [46]. One researcher verified all transcripts against the original audio recordings. All data were anonymized and stored securely for use in scientific presentations and publications.

### 2.5. Data Analysis

The interviews ranged from 17 to 46 min (mean = 27 min; SD = 6.87 min). The overarching data analysis followed the six-step approach outlined by Braun and Clarke (2006) [47]. The preliminary stage involved a period of familiarization to the data, which preceded the coding process. The transcription of interviews was conducted using f4X (2024) [46] to ensure the accuracy of the data. The second step involved initial coding, where two independent researchers (TL and PW) performed open coding on thirteen of 25 interviews using the method proposed by Kuckartz (2022) [42] as guidance for the structured analysis.

**Figure 1:**
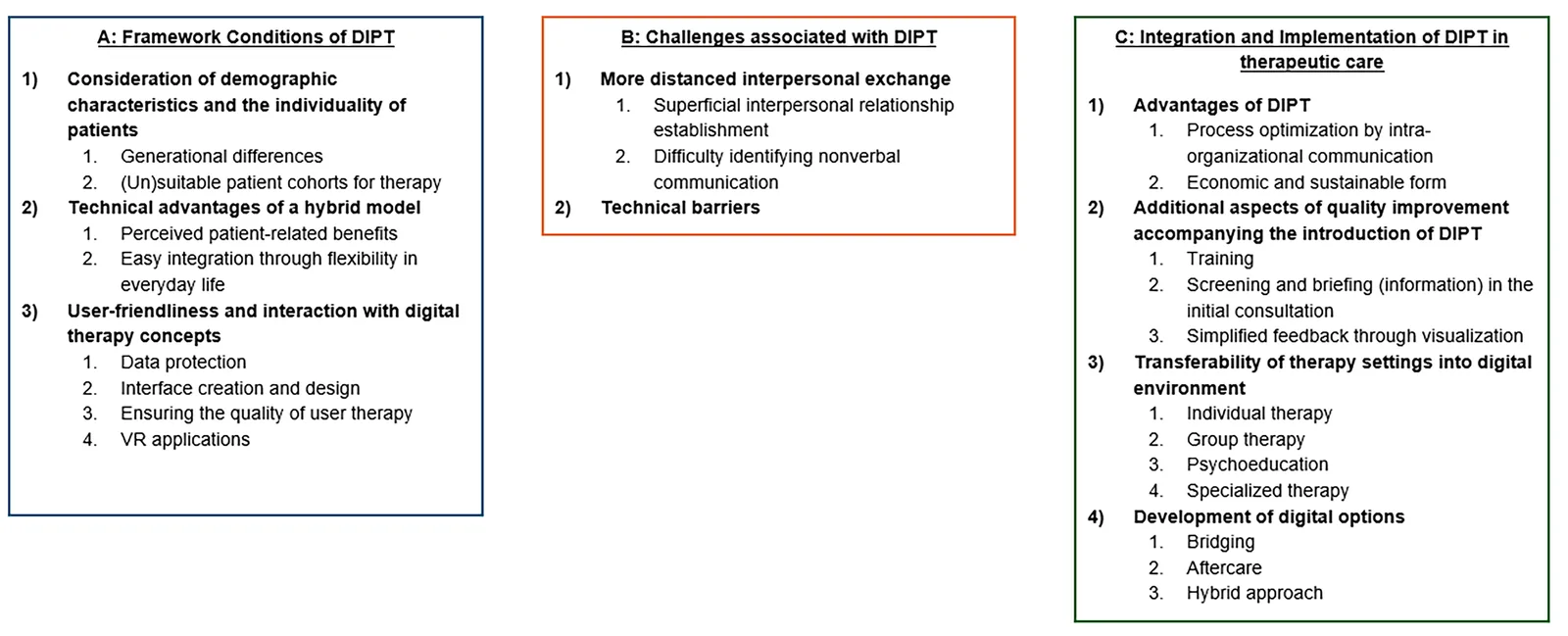
Codebook of thematic analysis.

The third step involved organizing and reviewing the initial codes generated by the two researchers (TL and PW) to identify potential themes. In the fourth step, a third independent coder reviewed six interviews to identify potential themes. Any discrepancies were resolved through discussion among the coders. In the second step, TL and PW discussed the discrepancies. Following the third step, SB and PW discussed the remaining discrepancies, with TL involved as needed. The researchers TL, SB, and PW engaged in a series of meetings and discussions to reach a consensus on the matter. After a thorough and deliberate process, the coders reached a consensus on the final codebook (see Figure 1). The finalized codebook was subsequently employed in the analysis of all interviews using MAXQDA (2024) [48]. Reported frequencies are descriptive of the present sample only and should not be interpreted as prevalence estimates or generalized to the wider population of MHPs. Following the completion of the codebook, PW undertook the coding of all 25 interviews, with 13 of these being subsequently cross-checked by TL. The basis of the hybrid deductive–inductive approach was the questions from the semi-structured questionnaire, and the codebook was created in an iterative coding process with the other coders. Subsequently, the interviews and codes were translated from German to English by PW to ensure accessibility for an international scientific audience. Thereafter, the research team reviewed the translations, and a bilingual team member checked them for semantic equivalence.

## 3. Results

The study included 25 participants (20 female, 5 male) aged between 24 and 64 years. The mean age was 37.32 years (SD = 12.06). Seven participants had leadership positions. A detailed overview of sociodemographic data and work-related characteristics of the participants is presented in Table 1.

When asked to estimate the amount of time they spent using digital media during a typical working day, five respondents reported spending less than three hours on digital media-related tasks, while ten respondents reported spending three to four hours on such activities. The remaining ten participants reported working with digital media for more than four hours during a typical workday (mean = 4.56, minimum = 2, maximum = 7, SD = 1.73). All participants reported using digital media on a daily basis during a typical working day (25 of 25 participants, 100%).

In response to the inquiry regarding the respondents’ familiarity with the concept of DIPT, the participants were first prompted to articulate their own interpretations or conceptualizations of the term. Following the provision of their own description, the participants were presented with the standardized definition of DIPT utilized in this study. A total of twenty-four participants provided descriptions that reflected the majority of the core elements of the study definition. However, one participant was unable to provide a clear description of the concept. The Clinic for Psychosomatic Medicine and Psychotherapy at the LVR-University Hospital Essen has conducted extensive research in the field of digitalization. The findings in this study reflect participants’ ability to describe or infer the concept and do not indicate documented prior exposure to DIPT, as prior exposure was not systematically assessed.

### 3.1. Cross-Professional Perspectives

The professional groups expressed generally positive views on digitalization but differed in their priorities. Physicians, like nurses, were concerned about the additional workload associated with digitalization and the resulting reduction in time for patient care. They hoped that digitalization would facilitate easy access to patient data, an expectation also expressed by the social worker. Nurses, in contrast, emphasized patient monitoring and practical workflow-related aspects. Psychologists and psychotherapists were concerned about potential interpersonal distance resulting from the loss of nonverbal cues in digital interactions, which could affect the therapeutic alliance. The specialist questioned whether digital therapy would be as effective as in-person sessions and expressed concerns about the potential loss of group cohesion, particularly in specialized therapies. The nutritionist highlighted the benefits of digitalization, particularly the easier and more effective provision of informational materials, which patients can also review repeatedly.

### 3.2. Structured Themes

The thematic analysis identified three overarching themes: (A) framework conditions of DIPT, comprising three themes focusing on the prerequisites for the implementation of DIPT, (B) challenges associated with DIPT, with two themes focusing on perceived limitations and risks, and (C) integration and implementation of DIPT in therapeutic care, comprising four themes focusing on future perspectives and opportunities for optimization. The overarching themes, themes, and subthemes are presented in Figure 1. All quotes cited in this section can be found in Appendix B.

### 3.3. Overarching Theme A: Framework Conditions of DIPT

**Theme 1: Consideration of demographic characteristics and the individuality of patients**

*Subtheme 1.1: Generational differences*

Ten participants noted that younger individuals tend to be more technologically competent. They emphasized the need for digital inclusion and alternative treatment approaches for patients with limited digital skills (see quotes (1–4)).

*Subtheme 1.2: (Un)suitable patient cohorts for therapy*

Several participants reported no major limitations regarding the use of digital therapy (see quote (5)). Participants identified several potentially suitable groups, including monosymptomatic patients, therapy-experienced individuals seeking a “refresher,” individuals with anxiety disorders, and those with mild to moderate depression. Other suitable groups include patients with eating disorders, such as binge eating, anorexia, and bulimia, as well as those with obesity, psycho-oncology patients, and trauma patients (see quote (6)). Participants emphasized that suitable patients should be assessed based on their sociodemographic characteristics and digital literacy.

In contrast, twelve participants identified patients with acute psychiatric conditions, or clinical instability as unsuitable, particularly in cases of acute suicidality. Other concerns included severe eating disorders, trauma, depression, anxiety, and patients requiring close monitoring (see quotes (7; 8)).

**Theme 2: Technical advantages of a hybrid model**

*Subtheme 2.1: Perceived patient-related benefits*

Participants emphasized the importance of improved access to care, particularly for patients in rural areas, those with limited mobility, and those experiencing social isolation. One participant remarked, “that’s why I think it’s essential for their care” (see quote (9)). Participants also associated DIPT with greater flexibility, treatment adherence, and self-efficacy. Virtual tours and introductions were suggested to help patients acclimate to the treatment setting (see quote (10)). Participants described self-monitoring functions and the integration of therapy into the home environment as potentially empowering for patients, citing these factors as an advantage of DIPT (see quotes (11–13)). However, they also identified the following concerns: limited self-responsibility, social withdrawal, symptom fixation, and varying motivation (see quotes (14–23)).

*Subtheme 2.2: Easy integration through flexibility in everyday life*

Fifteen of the 25 participants emphasized the ease of integration, citing the flexibility that DIPT offers in everyday life. Participants reported that DIPT could be integrated into patients’ daily routines and accommodate responsibilities such as childcare, work, and pet care (see quotes (24; 25)). The interviews highlighted that “digital treatment is an opportunity for more flexibility,” which can enhance participation (see quote (26)).

**Theme 3: User-friendliness and interaction with digital therapy concepts**

*Subtheme 3.1: Data protection*

“Data protection is also very important for the patients, that it is made transparent, that no data gets into unauthorized hands” (see quote (27)). Most participants identified data protection as a major concern. Transparent communication regarding data access rights with both internal and external clinical staff was emphasized as important for building patients’ trust (see quotes (28–30)).

*Subtheme 3.2: Interface creation and design*

Participants emphasized the importance of user-friendly and calming visual interfaces while avoiding aggressive color schemes and preferred interfaces resembling clinical environments rather than a call-center interface for patients (see quotes (31–33)). Additionally, participants expressed the need for simple language and intuitive design (see quote (34)).

*Subtheme 3.3: Ensuring the quality of user therapy*

Participants emphasized the need for structure, clear rules, consistent points of contact, and user-friendly functionality. Factors included simple language and operation, “so that everyone is on board” (see quote (38)). Adequate technical skills, implementation strategies, appropriate hardware and software, and ongoing IT support were considered essential for maintaining treatment quality and preventing digital overload such as excessive push notifications or chat access (see quotes (35–45)).

*Subtheme 3.4: VR applications*

VR tools were identified as useful for exposure therapy and orientation tasks, such as virtual-assisted grocery shopping or guided clinical tours (see quotes (46–48)). However, concerns were raised about limited social interaction, technical unreliability, and underdeveloped infrastructure (see quotes (49; 50)).

**Overarching theme B: Challenges associated with DIPT**

**Theme 1: More distanced interpersonal exchange**

*Subtheme 1.1: Superficial interpersonal relationship establishment*

Fourteen participants reported difficulty forming therapeutic relationships in digital settings. They described reduced emotional depth, trust, and motivation (see quotes (51–56)). Participants also described the change in therapeutic environment as an important and effective therapeutic factor in creating a sense of safety. Participants expressed the importance of face-to-face interaction on site. While some emphasized that interpersonal dynamics can only be “built in person” (see quote (57)), others noted that therapeutic relationships can be established in digital settings (see quote (58)).

*Subtheme 1.2: Difficulty identifying nonverbal communication*

Participants reported difficulties interpreting nonverbal communication such as facial expressions and body language via digital formats (see quotes (59–61)). Participants described strategies to mitigate the reduced availability of nonverbal cues in digital therapy. Participants suggested developing strategies for interpreting patients’ behavior in the digital setting, while prior face-to-face contact was considered beneficial for assessing patients during subsequent online sessions (see quote (62)).

**Theme 2: Technical barriers**

Common concerns included poor internet connectivity, insufficient devices and IT infrastructure, and outdated hardware and software. Participants emphasized the need for comprehensive handbooks and regular staff training to maintain therapeutic quality (see quotes (63–65)).

**Overarching theme C: Integration and Implementation of DIPT in therapeutic care**

**Theme 1: Advantages of DIPT**

*Subtheme 1.1: Process optimization by intra-organizational communication*

Participants recommended integrating the DIPT into hospital information systems (KIS), internal communication tools, digital documentation, online consultation forms, and billing systems. They expected the integration to simplify registration, enhance communication, and facilitate access to patient information for both clinical and research purposes (see quotes (66–71)). While a few participants doubted the feasibility of virtual ward rounds, others supported digital meetings and team coordination (see quotes (72; 73)).

*Subtheme 1.2: Economic andsustainable form*

Participants advocated reducing paper use by utilizing digital diaries (e.g., food-, sleep-, and symptom diaries) and digital forms (see quote (74)). The automated digital delivery of materials to patients was viewed positively (see quotes (75–77)). It was also mentioned that efficient scheduling, direct digital documentation, and quieter therapy environments could contribute to time and cost savings (see quotes (78; 79)).

**Theme 2: Additional aspects of quality improvement accompanying the introduction of DIPT**

*Subtheme 2.1: Training*

“I think training is always good when it comes to improving patient care in some way” (see quote (80)). Nearly all participants emphasized the need for technical training, particularly for staff with limited digital skills, as well as training in recognizing nonverbal cues in digital settings. While some favored learning-by-doing, others highlighted insufficient training and support for MHPs (see quotes (81–86)).

*Subtheme 2.2: Screening and briefing (information) in the initial consultation*

Participants recommended an initial, in-person consultation to assess patients’ digital literacy and overall suitability for DIPT (see quotes (87;88)). One emphasized the importance of this screening to determine “which patients are good candidates for the inpatient-like care and which patients like a digital clinical approach” (see quote (89)). An additional perspective was the suggestion to conclude with an in-person session to complete the therapeutic loop.

*Subtheme 2.3: Simplified feedback through visualization*

Three participants described the benefits of using visual elements, such as charts and diaries, to enhance patient engagement and understanding (see quotes (90; 91)). Tools like PROMSPACE (a hospital-internal patient management system) were cited as effective for delivering real-time feedback and increasing treatment transparency (see quote (92)). Participants suggested integrating visible feedback results from questionnaires, diaries, and monitoring tools to facilitate progress tracking and motivate patients by making success tangible.

**Theme 3: Transferability of therapy settings into a digital environment**

The present theme concerns the feasibility of various forms of therapy in a digital setting. Many stated that all types of therapy could be delivered digitally, emphasizing the importance of aligning digital formats with the specific goals and methods of each therapy (see quote (93)). Therapies identified as suitable for digital delivery included mindfulness exercises, relaxation techniques, skills training, nutritional protocols, diaries, cooking classes, and a range of other practices, such as family discussions, stabilization and imagination exercises, shopping training, and self-efficacy exercises (see quotes (94–100)).

*Subtheme 3.1: Individual therapy*

Nearly half of the participants viewed individual therapy as transferable to digital settings (see quotes (101; 102)). A smaller proportion expressed skepticism, particularly concerning the therapeutic depth achieved in the digital environment. Twenty participants had either delivered or could envision delivering individual therapy in digital form.

*Subtheme 3.2: Group therapy*

The participants’ opinions on this subtheme were not unanimous. Practitioners expressed support for online group therapy that had been preceded by prior in-person interactions. Conversely, others noted a decline in group cohesion and dynamics (see quotes (103–106)).

*Subtheme 3.3: Psychoeducation*

Half of the participants identified the effectiveness of digital psychoeducation for both patients and their relatives. They emphasized that digital modules and group sessions could be flexibly accessed and revisited when needed (see quotes (107–109)).

*Subtheme 3.4: Specialized therapy*

Specialized interventions, such as art therapy, neurofeedback, laughter yoga, and sports therapy, were viewed more critically. Seven participants felt these modalities were less suited to digital formats due to their reliance on physical presence, body awareness, materials, or group dynamics (see quotes (110–113)). Some participants suggested partial digitalization, such as digital warm-ups or instructional videos, as a compromise for digital movement therapy (see quote (114)).

**Theme 4: Development of digital options**

*Subtheme 4.1: Bridging*

A few participants emphasized the potential of using DIPT to bridge waiting times between diagnosis and therapy. It should serve as preparation for the actual therapeutic process, incorporating components such as psychoeducation, digital therapy sessions, symptom tracking, and digital preparation for upcoming treatments. As one participant noted, this “might be more time-efficient to bridge the gap” (see quotes (115–118)).

*Subtheme 4.2: Aftercare*

Aftercare is a crucial component of digital treatment support. It helps patients transition back to their daily lives after treatment and focuses on relapse prevention. Participants advocated for the use of DIPT in aftercare settings, viewing it as especially beneficial for patients with limited access to in-person follow-ups (see quotes (119; 120)).

*Subtheme 4.3: Hybrid approach*

More than half of participants supported a hybrid care model that combines digital and face-to-face treatment (outpatient or inpatient) as an optimal long-term approach. Participants emphasized that digital therapy “… can’t completely replace a ward, but it can complement it well” (see quotes (121–125)). One participant summarized this vision, stating, “I believe that everyone would benefit if we were to combine the best of both worlds, replace certain things, and perhaps create more room for other things as a result” (see quote (126)).

## 4. Discussion

The aim of this study was to explore the attitudes and expectations of MHPs concerning the DIPT concept through semi-structured interviews. The analysis identified three primary overarching themes from the data: (A) the framework conditions of DIPT, (B) challenges associated with DIPT, and (C) the integration and implementation of DIPT in therapeutic care.

The findings suggest that MHPs generally have positive attitudes toward DIPT, although these attitudes were accompanied by concerns and reservations. Concerns also relate to the comparatively slow digitalization of the German healthcare system. In countries such as Estonia and the Netherlands, telemedicine and video consultations are already well established as part of routine healthcare [18,27]. Another practical challenge is the law passed in 2026 to stabilize contribution rates in the statutory health insurance system, which is intended to result in cost savings in the healthcare sector. These savings may also affect psychotherapy [49]. At this juncture, the short-term and long-term ramifications remain indeterminate, primarily due to the recent implementation of these modifications.

Differences in perceptions of digitalization were observed across professional groups. Digitalization was regarded as a means of improving access to information and efficiency, but concerns remained regarding workloads and interpersonal and therapeutic interactions. The process of technology adoption can be comprehensively understood through the lens of the UTAUT framework [36], which elucidates the factors that influence users’ acceptance and adoption of technology. Some MHP groups have emphasized on performance expectancy, including such factors as improved efficiency and access to information. Conversely, other studies have placed greater emphasis on effort expectancy and facilitating conditions, including workload, usability, technical infrastructure, and training. The role of social influence was evident in the value assigned to interdisciplinary communication and organizational support. In this context, DIPT has the potential to support MHPs by facilitating administrative processes, enhancing workflow integration, and potentially liberating time for direct patient care. Therefore, it can be posited that usability, adequate infrastructure, and proper training may be important prerequisites for successful implementation.

Other studies [28,29,30] supported the suggestion that DIPT may be particularly relevant for underserved populations with limited access to psychotherapy, particularly in rural areas. DIPT may also help patients better integrate therapy into their daily routines, thereby enhancing self-efficacy and self-monitoring. Furthermore, DIPT may have the potential to alleviate pressure on limited human resources and healthcare infrastructure, a particularly relevant advantage in light of the ongoing and projected shortages in both MHPs and information technology personnel [15].

Participants emphasized the importance of comprehensive eligibility assessments during the initial consultation. These assessments appear important for evaluating patients‘ clinical stability, technical competence, and motivation to engage in digital therapy. They also support the selection of appropriate treatment models, such as a bridging model to reduce waiting times, or a hybrid model that combines digital and in-person sessions.

A review of the existing literature by Fuhr and colleagues supports the hybrid model as the preferred approach, emphasizing its role in complementing, rather than replacing, face-to-face therapy [50]. Hybrid approaches have demonstrated the potential to reduce attrition rates and enhance therapeutic outcomes [33], a sentiment echoed by the participants and supported by findings in other literature [34,50]. Although digital interventions may not fully replace traditional face-to-face therapy in all clinical contexts, they can provide meaningful supplementary benefits [19,34,51]. Furthermore, DIPT may serve as a long-term support tool, particularly in aftercare settings, by fostering continuity and reducing the risk of relapse.

While individual therapy is generally considered well-suited for digital delivery, participants raised concerns about the efficacy of group therapy in virtual environments. Difficulties include managing group dynamics and fostering emotional connection. To address these issues, some suggested that group members meet in person before beginning digital sessions to build rapport and cohesion. Nevertheless, evidence suggests that group dynamics can indeed develop in online settings [52].

Specialized therapies involving movement, physical activity, or creative expression were generally perceived as less suitable for digital delivery because they rely on sensory experiences and physical presence. However, some components, such as psychoeducation, skills training, and self-monitoring, can be effectively digitized to improve treatment adherence and patient empowerment [28]. In this study, participants, for example, mentioned modalities that included mindfulness and relaxation exercises, diaries or dietary protocols, and nutritional therapies, as potentially suitable for digital delivery. VR applications were highlighted as a potential tool for exposure therapy, shopping training, and observational tasks [22,23].

Another challenge of digital therapy identified by participants was the reduced ability to observe nonverbal cues [53]. These limitations may be partially mitigated through appropriate technical setups (e.g., full-body camera visibility), as well as through novel strategies and training, before and during digital intervention [30]. Additionally, clear guidelines regarding privacy, the therapeutic environment, and appropriate technical conditions should be established and discussed with patients before treatment [54]. Leukhardt et al. (2021) [55] showed that one can establish and maintain a therapeutic relationship in a similar manner. Other studies that compared digital therapy and traditional face-to-face therapy found no difference in therapeutic outcome [51,56]. Participants also underscored the necessity of robust technical infrastructure, including reliable internet connectivity, the utilization of contemporary hardware and software, and ensuring software compatibility for both patients and providers. Furthermore, ongoing IT support, equitable access to digital tools, and secure data management were identified as prerequisites for implementation. These concerns align with previous findings that emphasized infrastructure [34,51,54,57] and data security [28,54,58,59] as significant challenges to implementation. A recent national survey found that 76% of hospitals in Germany reported IT workforce shortages, further hindering digitalization efforts [15]. Another frequently mentioned concern was the potential for a more superficial therapeutic relationship between patient and practitioner. Participants expressed that digital media may hinder the development of a strong therapeutic alliance, potentially resulting in a more impersonal and emotionally distant experience. The quality of the therapeutic alliance, resulting in a more impersonal and emotionally distant experience. The quality of the therapeutic alliance has been shown to be associated with treatment outcomes [20,60].

The motivation and acceptance of DIPT among MHPs may increase if they receive adequate training and active involvement in the implementation process, a willingness reflected by participants in this and other studies [28,61]. Nevertheless, according to Baumann et al. (2021) [37], resistance among staff is seen as a major barrier. Therefore, implementation strategies should also address staff workload, job satisfaction, and organizational support. Effective implementation strategies are essential to avoid negative consequences such as increased workload [28,61].

The DIPT platform should emphasize user-friendliness by incorporating clear language and intuitive interface design [62] that mirrors a clinical setting. Calm and approachable visual esthetics can further enhance the user experience. The implementation of regional cooperation models has been shown to improve the integration of hospital information systems and reduce fragmentation [63]. Participants also emphasized the importance of intra-organizational communication tools to optimize internal workflow, consistent with findings from previous studies [17,27]. Furthermore, DIPT should incorporate appropriate hardware and software capable of capturing nonverbal communication and adhering to structured protocols to maintain therapeutic quality. Banbury et al. (2018) [52] found that prior digital inexperience does not significantly impede usability, and participants reported improved comfort over time. Overall, successful implementation of DIPT will require alignment with clinical standards, flexibility in treatment formats, and support for interdisciplinary collaboration.

From a healthcare systems perspective, DIPT could help reduce waiting times, facilitate aftercare, and provide effective bridging interventions [18,57,64]. Digital therapies also enable continuous monitoring, which may help prevent rehospitalization and improve care continuity [10]. However, ethical and legal challenges, particularly those related to data protection, remain a critical concern [18,34]. Transparent data handling, secure access protocols, and robust data protection measures are important for maintaining trust between patients and providers.

Uncertainty persists regarding the appropriateness of DIPT for specific patient groups. Participants perceived DIPT as potentially less suitable for individuals with acute psychiatric conditions, such as psychosis, schizophrenia, substance use disorders, or clinical instability. However, opinions varied regarding the suitability of DIPT for conditions such as depression and eating disorders, despite growing empirical evidence for digital interventions in these populations. This highlights the need for further empirical research.

Previous studies on DIPT have shown that students generally have positive attitudes toward digital mental health solutions and are open to them, while also recognizing challenges associated with digitalization. Similarly, patients tend to favor hybrid approaches combining digital interventions with professional support. Overall, stakeholder priorities differed. Patients emphasized accessibility, MHPs focused on safety and quality, and students highlighted the importance of professional support alongside digital solutions. Including both students and patients‘ perspectives provides valuable insights into the expectations of future MHPs and the needs of end users, supporting the development of accessible and user-centered DIPT.

### Limitations

This study has several limitations. First, the qualitative research design is inherently context-specific [65], which limits the generalizability of the findings. Second, there is a potential for sample bias and institutional allegiance, as participants were recruited from a university hospital with a strong research focus. They may therefore have held more positive attitudes toward digitalization than professionals working in other healthcare settings. Additionally, not all professional groups involved in patient care were equally represented, with some groups being included only sporadically. The voluntary nature of participation may have introduced a degree of self-selection bias, potentially limiting the representativeness of the sample. Moreover, courtesy bias cannot be entirely excluded as participants may have been inclined to provide a more favorable response.

Furthermore, the standardized description of DIPT provided to participants prior to the interviews may have introduced a framing effect or confirmation bias. The questions included several positively worded descriptions of DIPT, and some interview questions contained leading information, which may have influenced participants’ responses. Therefore, the findings should be interpreted with caution, as participants’ reported attitudes may have been influenced by the way DIPT was presented during the interviews. Overall, the study examined attitudes and expectations regarding a theoretical model of DIPT. Consequently, the findings do not provide evidence regarding its implementation, feasibility, effectiveness, safety, or cost-effectiveness.

Finally, because the study was conducted at a single urban hospital, the findings may not be applicable to other healthcare settings and populations. To enhance the external validity and generalizability of the results, future research should therefore include a broader range of institutions, professional groups, and geographical regions.

*Implications for practice and policy:*

DIPT may offer opportunities to improve access to psychosomatic care and potentially reduce waiting times if implemented in clinical practice. However, the challenges described above regarding the therapeutic relationship between patients and MHPs and digital literacy could arise.

DIPT could be used as a hybrid care model to facilitate integration into everyday clinical practice, while taking patient suitability into account.

In order to implement digital psychotherapy and psychosomatic care in a sustainable and evidence-based manner, health policy stakeholders must first establish quality standards, infrastructure, and reimbursement models.

## 5. Conclusions

Overall, participants expressed positive attitudes toward the DIPT concept. MHPs expressed a desire for easily accessible, comprehensive, flexible, multimodal, and interdisciplinary treatment options. However, successful implementation depends on several critical framework conditions. These include ensuring stable internet connectivity, clarifying technical support structures, enhancing digital competence among MHPs, securing appropriate hardware and software, and providing regular training. In addition, it is essential to define suitable patient cohorts and establish a clear implementation framework that aligns with the therapeutic goals of specific clinical contexts. The findings suggest that these foundational elements may be important prerequisites for the effective implementation of DIPT. A hybrid care model may help preserve and strengthen the therapeutic alliance while allowing patients to benefit from the flexibility of digital treatment solutions.

## Figures and Tables

**Table 1:** Sociodemographic data and work-related characteristics.

Characteristics	Values	
	*n*	%
Years of employment, in years		
<1	5	20
1–3	7	28
3–5	5	20
>5	8	32
Confidence in using current digital technologies		
Very good	16	64
Good	9	36
Primary use of media at work *		
Training courses	15	60
Hospital information system	14	56
Online meetings	14	56
Microsoft Office	13	52
Clinical workflow management	12	48
Statistics	12	48
Therapy-related task (e.g., planning, implementation)	7	28
Internet browser	5	20
Video conferencing software	3	12
Work schedule planning and employee self-service	6	24
Medication program	3	12
Quality management	2	8
Questionnaires	2	8
Digital health applications	2	8
Use of digital media for therapeutic purposes		
Yes	21	84
No	4	16
Frequency of use		
Daily	3	12
Once a week	2	8
Several times a week	4	16
Several times a month	10	40
Rare	2	8

Note: *n* = 25. * = Multiple responses were possible; therefore, percentages do not sum to 100%.

## Data Availability

The data supporting the presented results are available from the corresponding author upon reasonable request due to privacy restrictions.

## Abbreviations

The following abbreviations are used in this manuscript:

AMBI	outpatient intensive complex treatment
APAH	acute psychiatric treatment at home
BPtK	Bundes Psychotherapeuten Kammer (Federal Chamber of Psychotherapists in Germany)
COREQ	Consolidated Criteria for Reporting Qualitative Research
DiGA	digital health applications
DIPT	digital inpatient-like psychotherapy
e-PA	electronic health records
e-Rezept	electronic prescriptions
iCBT	Online Cognitive Behavioral therapy
KIS	hospital information systems
MHP	mental health professional
StäB	inpatient-equivalent psychiatric treatment
UTAUT	Unified Theory of Acceptance and Use of Technology
VR	virtual reality
WHO	World Health Organization

## Appendix A

### Appendix A.1. Guidelines

**Sociodemographic data:**

1.	Interview number				
2.	Age				
3.	Gender	female □	men □		diverse □
4.	What is your primary function at the LVR-Klinikum Essen?	Assistant doctor		☐	
		Medical specialist		☐	
		nurse		☐	
		Psychologist		☐	
		Psychotherapist		☐	
		others		☐	
5.	Do you have an leading ship position?	Yes		☐	
		No		☐	
6.	How long have you been employed at the LVR Clinic in Essen?	<1 year		☐	
		1–3 years		☐	
		3–5 years		☐	
		>5 years		☐	

**Frequency of use of digital media in their profession:**

1.	How well do you cope with current digital technologies?	Very good	☐
		Good	☐
		Less good	☐
		Not at all	☐
2.	How often do you use digital media in your normal working day?	Daily	☐
		Once a week	☐
		Several times a week	☐
		Several times a month	☐
		Rare	☐
		Never	☐
3.	Estimate your workload with digital media in hours on a normal working day. (Please indicate approximate number of hours)		
4.	For what purposes do you currently use digital media primarily in your day-to-day work? (Feel free to name programs and other activities)		
5.	Have you ever used digital media for therapeutic purposes in your everyday work?	Yes	☐
		No	☐
5a.	If so, how regularly?	Daily	☐
		Once a week	☐
		Several times a week	☐
		Several times a month	☐
		Rare	☐

**Familiarity with the concept of digital inpatient-like psychotherapy:**

1.	Are you familiar with the concept of “digital inpatient-like psychotherapy”?	Yes	☐
		No	☐
	No: Can you imagine what that might mean? (then continue with the explanation)		
	Yes: Can you explain what the term means to you? What comes to mind spontaneously?		

**Digital inpatient-like psychotherapy—explanation:**

As digitalization continues to advance in the healthcare sector, both the opportunities and the requirements for patient-oriented, comprehensive, and individual treatment of people with mental illness are increasing. The innovative concept of DIPT is intended to enable patients and caregivers to discover and make the best possible use of these new opportunities.

DIPT offers the possibility of low-threshold and flexible multimodal and multiprofessional treatment in a digital environment. Due to its spatial independence, it is accessible without restriction—also for external practitioners (experts), barrier-free and enables individualized treatment intensity.

In addition to digital diagnostics, video consultations, and home-monitoring methods (e.g., wearables and mobile biofeedback), patients have the opportunity to flexibly collocate therapy components, integrate them into their daily lives, and actively shape their treatment. This not only strengthens the sense of autonomy but also the responsibility for their own therapy.

Evidence-based psychotherapeutic procedures (e.g., Internet-based cognitive behavioral therapy) are provided via video in sequential individual and group treatments, which are complemented by selected psychotherapy apps (e.g., with interactive exercises, videos, audios for psychoeducation, mindfulness, etc.).

The primary point of contact for all functionalities and offerings is an intuitively usable/structured clinic app. Through the use of virtual reality, digital treatment is made experienceable for patients and providers and brings them together.

The digital clinic, as an interface between inpatient-like and outpatient therapy, thus enables an individualized addition and further development in the sense of holistic patient care.

**Qualitative Questions:**

Attitude	1. What do you think of digitalization in medicine and especially in psychosomatic medicine and psychotherapy?
	2. What is your attitude toward a digital station?
	3. Do you generally feel comfortable using digital technologies/media (technologies: smartphone, tablet, computer, laptop; media: app, website, forum …)?
	4. Could you please share your thoughts and reflections on how digital media could support your day-to-day work in some way?
	5. Are you familiar with digital methods that can be used to supplement therapeutic work (e.g., apps) and do you use such methods in your day-to-day work?
	6. Can you identify possible disadvantages or challenges that could arise in connection with the introduction of a digital treatment concept?
	7. Can you imagine digital services helping patients in their everyday lives in the same way as conventional therapy methods?
	8. Do you think that digital services could replace personal contact? Do you see this as positive or negative?
Perception	9. Which patient group would benefit most from a digital offering?
	10. Are there patient groups that should NOT benefit from or participate in a digital service? Why?
	11. What do you think a DIPT should look like and what digital options should it include?
	12. What are your concerns about using such a DIPT?
	13. Is there any important information that you as a practitioner would need to see in a DIPT (ward rounds, laboratory values, medication plan, etc.)?
	14. What digital interventions can you imagine and what could they look like? (concrete exercises such as individual therapy, group therapy, art therapy, music therapy, etc.)
	15. In your opinion, are there any interventions that should NOT be offered digitally?
	16. Do you think it is necessary to provide training for the practitioners of these interventions before they start using them? (Instruction on the program, special requirements such as training for non-verbal gestures—as the practitioners have a harder time assessing the patients during the interventions as they are not face-to-face)
	17. Do you think that digital treatment approaches might be missing important elements or aspects that are present in traditional face-to-face treatment? If so, could you please elaborate on what these aspects might be and how this might affect the treatment?

**Closing Question:**

1.	Do you have any other comments on this topic?
2.	Tell me the three most important points that I should take with me

### Appendix A.2. Consolidated Criteria for Reporting Qualitative Research (COREQ)

**Domain 1: Research team and reflexivity** Personal characteristics	
I. Facilitator	Patrick Jonas Wollenberg
II. Credentials	Patrick Jonas Wollenberg (doctoral student)
III. Occupation	**Research associate:** Lucy Ann Gresser, Patrick Wollenberg, Rebekka Robitzsch, Tania Lalgi, Christoph Jansen **Postdoctoral researcher:** Alexander Diel, Anita Robitzsch, Alexander Bäuerle **Full-time professor:** Martin Teufel
IV. Gender	Female: TL, LG, RR, AR Male: PW, CJ, AD, MT, AB
V. Experience and training	**PW:** human medicine student, doctoral student **TL:** educational background in psychology (M.Sc.), practical experience in qualitative research **LG:** human medicine student, doctoral student **CJ:** senior psychosomatics physician **RR:** assistant psychosomatics physician **AD:** postdoctoral researcher **MT:** chief psychosomatics physician and professor **AB:** educational background in psychology (M.Sc.), postdoctoral researcher, and head of research for psychosomatic medicine and psychotherapy **AR:** senior psychosomatics physician, head of outpatient unit
***Relationship with participants***	
VI. Relationship established	PW had no prior personal or therapeutic relationship with the participants. Participants and members of the broader research team were affiliated with the same clinical institution (Clinic for Psychosomatic Medicine and Psychotherapy at the LVR-University Hospital Essen). Participation in the study was voluntary.
VII. Participant knowledge of facilitator	The participants in the present study knew that PW is a doctoral student of the institution
VIII. Facilitator characteristics	No other characteristics were reported about the facilitator
Domain 2: Study design	
**Theoretical framework**	
Methodological orientation and theory	Hybrid deductive–inductive codebook thematic analysis, informed by Braun and Clarke (2006) [47], with Kuckartz (2022) [42] used for structural guidance during coding and codebook development.
**Participant selection**	
Sampling	Recruitment took place between 27 March and 18 June 2024, and the interviews were conducted between 27 March and 17 July 2024, due to limited availability of some MHPs
Method of approach	Email and Flyer
Sample size	*n* = 25 participants
Non-participation	Sixteen declined or not responded, the reasons are unknown
**Setting**	
Setting of data collection	Twenty-four interviews were conducted in person at the LVR-University Hospital Essen. One interview was conducted online.
Presence of non-participants	None
Description of sample	Between 24 and 64 years (M = 37.32; SD = 12.0648), 20 female (80%) and 5 male (20%)
**Data collection**	
Interview guide	Provided as supplemental material: Appendix A.1, Appendix A.2 and Appendix B
Repeat interviews	None
Audio/visual recording	Audio recording
Field notes	Yes
Duration	17–46 min; (*M* = 27, *SD* = 6.87)
Data saturation	No new themes or subthemes emerged during the final interviews at the level of the overall dataset.
Transcripts returned	No
Domain 3: Analysis and findings	
Number of data coders	Three coders: PW, TL, and SB
Description of coding tree	Yes (final coding framework is presented in Figure 1.)
Derivation of themes	The following themes (A) Framework Conditions of the DIPT, (B) Challenges associated with DIPT, (C) Integration and Implementation of DIPT in therapeutic care were derived using a hybrid deductive–inductive approach. The research questions and interview domains functioned as an initial organizational framework. The codes and subthemes were developed inductively from the data and iteratively refined through consensus discussions.
Software	MAXQDA 2024 [48]
Participant checking	No
**Reporting**	
Quotations presented	Yes
Data and findings consistent	Yes
Clarity of major themes	Yes
Clarity of minor themes	Yes

## Appendix B

*Quotes for Themes and Subthemes*

**Overarching themes A: Framework Conditions of the DIPT**	
**Subtheme 1.1: Generational differences**	(1) I’m sure there are older people who can do it well. But it will probably be the case that younger patients in particular, who simply grow up with a completely different understanding of technology, will be able to use it more intuitively. (Interview 004 D, pos. 46) (2) I think you have…(to) make sure that you somehow reach people who perhaps have no experience in the digital field. (Interview 005, pos. 70) (3) …it makes a difference whether I have a team with an average age of 25 or 30 or a team with an average age of 50 to 60, because I think that’s how you grow up with social media. (Interview 003, pos. 48) (4) Of course, there are older patients who may not be able to cope with the media. In that case… you would have to offer the alternative in parallel. (Interview 006, pos. 68)
**Subtheme 1.2:** **(Un)suitable patient cohorts for therapy**	(5) So I think every disorder could benefit from a digital service. (Interview 015, pos. 57) (6) I would really say depressive patients, eating disorder patients, perhaps also traumatized patients, something like that. (Interview 013, pos. 48) (7) If we now look outside psychosomatics, i.e., in the field of psychiatry … I could imagine that it would be more difficult. Simply in the context of danger to self and others. I’m just thinking about acute psychoses …. (Interview 007, pos. 44) (8) Outpatient psychotherapy in video format… is contraindicated for severe eating disorders, complex traumatization, severe self-endangerment, etc. (Interview 022, pos. 44)
**Subtheme 2.1:** **Perceived patient-related benefits**	(9) Digital services are great additions and opportunities to reach people who would otherwise not be reached. And that’s why I think it’s essential for their care. (Interview 009, pos. 82) (10) Looks, I can also very well imagine a kind of virtual tour of a clinic to prepare patients for treatment in combination with brief introductions of the individual professional groups. (Interview 025, pos. 64). (11) …it would be something more low-threshold, one or two hours a day, where so much can be done independently. (Interview 003, pos. 66) (12) … how to make patients take responsibility for themselves… it would no longer be possible for me to measure blood pressure or blood sugar… perhaps the patient could enter their values themselves… in the platform… (Interview 005, pos. 64) (13) Many patients find it really difficult to detach themselves from this bubble after an inpatient-like stay and go home again… it’s really great if you can get therapy at home in your normal everyday life right from the start, because then you don’t go from 100 to zero… (Interview 013, pos. 68) (14) … the infrastructure is not there in such a way that they could come in on a mobile basis (Interview 005, pos. 32) (15) …I have to say that there are times, such as the recent pandemic, when it’s important to provide things that are location-independent and more flexible. (Interview 013, pos. 34) (16) … major obstacles to making personal contact, whether for reasons of illness or because they live in very rural areas and may not have a car (interview 008, pos. 50) (17) … it will relieve a lot of patients and simply make access to psychotherapy easier. (Interview 007, pos. 36) (18) …And patients who live in isolation. (Interview 017, pos. 64) (19) Therapies are then carried out from anywhere or from home… might be less effective than if you really have a place to go. (Interview 013, pos. 68) (20) …patients get a sense of self-efficacy through this digital clinic and have the feeling that I’m doing something. (Interview 013, pos. 54) (21) The patient’s self-efficacy depends on how much they use these digital tools (Interview 016, pos. 90) (22) But especially at the beginning, I think it’s difficult when the patient is not yet ready to take on so much personal responsibility (Interview 024, pos. 44) (23) I have all that and I get the therapy on my living room couch, so to speak. And that can of course be counterproductive in the end (interview 005, pos. 46)
**Subtheme 2.2:** **Easy integration through flexibility in everyday life**	(24) … someone who … need a lot of flexibility because I have five children at home, five dogs at home, … your life circumstances are arranged in this way, then I think that can work really well. And then I don’t think that’s necessarily a disadvantage, because it takes away stress (Interview 007, pos. 56) (25) …patients who are actually still working and can’t imagine missing six, eight or ten weeks… patients who have small children, who are caring for others, who simply cannot participate in such inpatient-like or outpatient services without digital media due to structural circumstances. (Interview 019, pos. 36) (26) Digital clinic an opportunity for more flexibility (Interview 019, pos. 83)
**Subtheme 3.1:** **Data protection**	(27) … data protection is also very important for the patients, that it is made transparent, that no data gets into unauthorized hands (Interview 008, pos. 42) (28) … there is always the question of who is allowed to see my content within the clinic? So is everyone from every department really allowed to see everything? I think that’s another question of access rights. Another big concern is, IT security. What about hacker attacks if a digital structure collapses, as it did here at the university. What happens if everything is lost? Or if there are somehow errors and it gets lost? (Interview 009, pos. 76) (29) I couldn’t imagine that the general practitioner would go there and simply have access to the pathway. So that would all have to be after the patient’s approval … so that the patient has a say (Interview 014, pos. 105) (30) …. So I don’t have access to all the content either. So I could also imagine that individual therapy is protected, that not everyone in the team can access it (Interview 014, pos. 107)
**Subtheme 3.2:** **Interface creation and design**	(31) … So user guidance plays an incredibly important role so that you don’t have so many questions, especially when you’re confronted with different levels of technological competence. (Interview 021, pos. 54) (32) No red color (Interview 014, pos. 97) (33) …. Somehow I don’t think it would be so nice if you were sitting in a call center. So just in boxes like that and yes, I don’t really have an idea of that (Interview 006, pos. 58) (34) … very simple user interfaces, which should of course be ergonomically designed (Interview 022, pos. 78)
**Subtheme 3.3:** **Ensuring the quality of user therapy**	(35) … I miss a certain seriousness… when patients take the call while they’re sitting in the car, driving to work, so to speak, to go shopping, I think there’s a certain commitment in somehow sitting down here together at 3 p.m. and taking an hour for psychotherapy. … (Interview 003, pos. 46) (36) There is a risk that you switch off your camera and then do something else (Interview 024 D, pos. 40) (37) Very few people have a good camera (Interview 012 D, pos. 58) (38) I think it’s important that the programs or the structure is simple and easy to understand so that everyone is on board. (Interview 001, pos. 106) (39) …a clear structure, like the day clinic or ward here, which has a very fixed timetable, so that it’s not like I do it in my own time, but that you’re really in this ward treatment. (Interview 015, pos. 69) (40) …it might simply be necessary to have 27/7 contact persons… it might not be so feasible from a therapeutic point of view, but… like a patient hotline or something like that, which is then somehow attached to the hospital. (Interview 007, pos. 54) (41) … A team that is convinced that this (DIPT) offers a corresponding benefit for the patient. (Interview 009, pos. 62) (42) What I have also experienced in many digitalization projects is always a bit like the technical onboarding of employees and participants or patients. And that is always a question of the software … and the rollout concept or the onboarding concept on the other. So it always has to be both. (Interview 021, pos. 54) (43) …good software you can definitely remove I would say 70 to 80% of the barriers. If you make it user-centered and make the development user-centered… There is also the user-centered content level, but I believe that this 80% can be achieved, … (Interview 021, pos. 56) (44) I think I would find it important that it’s not a channel that runs all the time from one side, but that it’s also somehow a bit like the one-to-one conversations, … (Interview 015, pos. 71) (45) And the desire for better equipment to make digital clinics possible. (Interview 019, pos. 83)
**Subtheme 3.4:** **VR applications**	(46) …VR glasses… could also use them for parts of the confrontation. … be it for social phobic… fear of heights and I know that it can also be used with lots of things like driving and animals and that it also has a good effect. I could well imagine that. (Interview 005, pos. 58) (47) … working with VR glasses or something, then you could also do accompanied shopping or something, for example, so that you can really see… how does someone walk through the supermarket? Where does your gaze linger? Is that now and could you talk about it and so on? … (Interview 004, pos. 60) (48) I can also very well imagine a kind of virtual tour of a clinic to prepare patients for treatment in combination with brief introductions of the individual professional groups… (Interview 025, pos. 64) (49) I think it’s just something different, even if we’re sitting there with VR and avatars and no idea why, I think humans per se are very social beings and need proper social interaction. (Interview 004, pos. 69) (50) The opportunity. To experience therapy tools directly, so to speak, to make them directly tangible. Of course, I know that there is also the possibility of virtual reality. But I don’t think we’re at the point yet where we can simulate reality, so to speak. (Interview 025, pos. 52)
**Overarching theme B: Challenges associated with DIPT**	
**Subtheme 1.1:** **Superficial interpersonal relationship Establishment**	(51) Of course, you shouldn’t neglect the fact that one factor… is the therapeutic relationship. I have many patients who simply benefit from coming here, from having such a protected space… I do believe that there are also many patients who would somehow lack the effective factor of the therapeutic relationship or the change of location. (Interview 013, pos. 36) (52) The visits, in other words this benevolent, appreciative, reflective interaction, that this could be missing… (Interview 015, pos. 87) (53) My concern is that human exchange suffers as a result and I don’t think it disappears, but that it suffers because the interactions, which are incredibly valuable for our work, are both diagnostic and therapeutic for us… (Interview 003, pos. 70) (54) On the one hand, I perhaps believe that, as with all digital services, a certain degree of interpersonal contact may be lost, simply because of the distance we have, that we perceive situations differently when they are present. … If you somehow have a meeting that is online, then you might be doing something else on the side. (Interview 004, pos. 52) (55) It goes further out there, and digitalization is the maximum, you’re completely out of it. And when we say it somehow depends on relationship work. Relationship work via digital doesn’t work. You have to say that I can communicate things, but the content of the relationship is very, very poor. … take the step in here, come into human contact and then experience themselves anew. (Interview 017, pos. 56) (56) How do I find the feeling that you develop for each other in the live situation? I have noticed that in video therapy because I am already doing that. I don’t think it’s the same. (Interview 009, pos. 48) (57) But I think the basis of trust first has to be built up in person and in person. (Interview 010, pos. 64) (58) And that there was a therapeutic process, there were developments, it was also experienced as supportive and good. And I think the question now is whether you can say that it would have been somehow better if it had always been facing to face… (Interview 021, pos. 62).
**Subtheme 1.2:** **Difficulty identifying nonverbal communication**	(59) I don’t get the nibbling because I only see the upper body as it is. So I lose a lot, I think. (Interview 002, pos. 44) (60) In psychotherapy, we work relatively often with a feeling. It’s a feeling, which means that sometimes it’s not so easy to operationalize what exactly is missing. Because if you think about it carefully, there’s actually less missing. It might be a bit of facial expression, it might also be a bit of gesture. So how do I position myself in relation to the person? So sometimes it’s also a certain kind of diagnostic, so someone comes into the room, sits down, and says here I am or is someone totally stealthy, which five times somehow asks to sit down now, so things like that are no longer possible. (Interview 003, pos. 46) (61) Nonverbal Keys Total. Because if I only see a person’s face and not their whole-body posture as they sit in front of me, then I simply don’t have any information. And that’s also mutual. I’m missing information from the patient, and I’m missing it from myself…(Interview 017, pos. 94) (62) If the program is good, I think the posture is natural, what you see someone always only up to half. So the hands are visually missing exactly. So that’s definitely something I think you have to get used to. You have to learn to read people differently and it’s often good to know them in person and then see them again online so that you can assess them. (Interview 012, pos. 50)
**Subtheme 2:** **Technical barriers**	(63) …would be important that there is somehow a stable Internet connection and good technology with a good system and program (Interview 019, pos. 62) (64) …technical requirements have to be met. So the current disadvantage is that the clinics are not yet equipped for this, the patients are not equipped for this. So I think patients should actually be provided with computer devices… a good camera etc. So they hang behind patients, hanging around them with their cell phones. That’s really, really awful, because then you always have a cell phone picture (Interview 012, pos. 58) (65) So, of course, whenever there are changes in the field of technology and digitalization, the first challenge is to make sure that everything is technically accessible to everyone and that it all works. But I think that is a conditional challenge. (Interview 007, pos. 64).
**Overarching theme C: Integration and Implementation of DIPT in therapeutic care**	
**Subtheme 1.1:** **Process optimization by intra-organizational communication**	(66) … if we had everything on one platform, because that would of course be good in the sense that you wouldn’t always have to search for everything first, the individual pieces of information from different programs et cetera pp. (Interview 005, pos. 66) (67) Basically, the provision of information and so really simply having more information about clients or about structures at hand and being able to access it more quickly and perhaps also being able to make changes in information systems immediately and not having to write it down again and then somehow make a post it, but simply in the situation where you need it. (Interview 021, pos. 94) (68) Maybe just a period of time where you can exchange ideas, just like here on the ward. …If I now imagine that I can’t go over to my colleague because he might not be sitting next to me in the digital clinic, then I would have to have some kind of function to communicate better than just by phone. (Interview 019, pos. 64) (69) … if everything is integrated into one program. Of course, it would be nice to only use one platform and not several… (Interview 005, pos. 64) (70) … one station works on it and the other station does it too and you don’t actually know because the interface is missing. … And perhaps the digital clinic could help a little with structuring. (Interview 004, pos. 64) (71) Yes, as much information as possible, because our experience in the outpatient clinic is that patients are often very incomplete in their explanations. So it’s often certainly not malicious to omit information, treatment, etc., but there’s often so much that they can’t keep it all straight. And if you have the hard facts and can rely on certain things and perhaps address them again, that helps a lot. So collecting all the information you can. (Interview 008, pos. 66) (72) I imagine ward rounds might be difficult. (Interview 015, pos. 99) (73) We would probably also network with each other in the digital clinic, so not just with patients, but also perhaps handovers or if everyone can share screens immediately, so that you have all the information immediately … that would be an immense relief … (Interview 005, pos. 66)
**Subtheme 1.2:** **Economic and sustainable form**	(74) … you now have relaxation apps or the documents for, … a recovery group or an empowerment group or a lot of paperwork that you always liked and still like, so that you outsource it and say okay, you get it all on your cell phone and you can look at it and maybe, if you work with a tablet, somehow make notes on it and then send it to yourself by e-mail …(Interview 006, pos. 64) (75) … no longer waste so much paper is a good thing, but it also means that things move much more quickly, and patients can access the material much faster. … I believe that all this really makes everyday life easier. (Interview 014, pos. 44) (76) If I could send the worksheets to the patients and they could also fill them out because they have an iPad, then that would perhaps be a relief because I wouldn’t have to run to the printer. We could save the paper, I could save the worksheets that I always do as favorites and the patients could call them up again later and not have to walk around with a mountain of paper… we also do diagnostics on paper from time to time. … Of course, that would also be practical if it were all online. Some of it is already online. But not all of the diagnostics we do. (Interview 019, pos. 72) (77) …. I’m just thinking about it, so it would also be cool if you could provide patients with information material or worksheets and things like that digitally. That would be great if you could somehow send them digitally or see what progress they’re making. So whether they actually work on the things and if so, I don’t know when they work on them, how they worked on them, that when you work with patients, you can provide them with any homework digitally and not somehow still work with sheets or something and then see how they worked on them or they can somehow ask questions or something… (Interview 013, pos. 64) (78) I’m more flexible in terms of working hours and where I work. I’m someone who also likes to work from home from time to time because, as I said, you have a room for it. Of course, you can see more patients in quick succession. More. Digitization is often also a process optimization. (Interview 012, pos. 66) (79) I can also be more productive as a therapist if necessary, I say, that’s also a bit, perhaps the introspection skills etc. (Interview 022, pos. 58)
**Subtheme 2.1:** **Training**	(80) … I think training is always good when it comes to improving patient care in some way. (Interview 024, pos. 74) (81) So training is essential in any case. I … say it’s not possible without it, it’s essential. (Interview 025, pos. 60) (82) I think it’s necessary for the sole reason that you can take advantage of the full variety of therapies on offer, that you’re well up to date. What can these video systems actually do? What possibilities are there? How can I perhaps rectify errors more quickly if they occur? Otherwise difficult. (Interview 009, pos. 50) (83) I think there was some communication training or something like that. So yes, with psychological psychotherapists, they learned how to communicate. But none of us have learned that in the age of we only do video calls or something like that. That’s why I think it’s really important to offer training or communication training for video calls or telephone calls etc. and also program training… (Interview 013, pos. 70) (84) … It’s called training or it’s somehow called familiarization. Some programs are so intuitive that you don’t need much training. PROMP Space was also a lot of learning by doing, but it was still good to know somehow (Interview 003, pos. 48) (85) So I’m not sure whether you necessarily need this non-verbal training for this gesture because of the integration of digital media, because I haven’t had the experience that you also have these transfer effects somehow, of course it hasn’t been investigated, but that would be my feeling. (Interview 021, pos. 92) (86) …little attention … paid to training elements for employees … sometimes I miss the training, and the support that comes with it (Interview 16, pos. 76)
**Subtheme 2.2:** **Screening and briefing (information) in the initial consultation**	(87) And here too, of course, you have to look at what kind of patients they are, how they deal with technology and their attitude toward it. Can you imagine that? Are they fit with such applications or are they … overwhelmed … and then frustrated and don’t use them anyway. … that will probably be a bit of a challenge, a challenge to really look at what is really needed now? So what makes sense now, even at this point? …. (Interview 004, pos. 44) (88) I think it would be great if there were patients who could make the journey, so that you could somehow get to know them in person at the beginning. Even in the group you start with or something if you like. But otherwise it’s definitely essential. At least a video call or a phone call where you really have some kind of contact… Nevertheless, at the beginning, you really need to know what diagnosis we are treating the patient with and is the diagnosis really confirmed? (Interview 013, pos. 56) (89) which patients are good candidates for the inpatient-like care and which patients like a digital clinical approach (Interview 13, pos. 66)
**Subtheme 2.3:** **Simplified feedback through visualization**	(90) … it would be good if it were displayed graphically, that you …have something like a focus that you can define together with the patient and also monitor a little. I think distress and sleep are not unimportant… If there are patients who somehow need skills, for example, then it would of course be good to see how many skills have been used or even symptoms. To show the symptom burden. Incident reporting, in other words. Bulimia Think vomiting. How often did I vomit? Something like that. (Interview 021, pos. 72) (91) And we also want to monitor and not only the outcome of therapy, but also the physical condition, mental condition in general, so somehow also the possibility. To regularly query certain parameters in a programmed way… (Interview 021, pos. 100) (92) Then I get the ROMS and then I get the questionnaire and then I look at the, I have that, so there is an incredible amount of psychometric data and there are so many tools, all the tools that already evaluate the ECG. Yes, the lab. Maybe it’s the same with psychometric diagnostics. I mean, we’re working on that, but I think that would be cool. That would also be really helpful for the practitioners. Helpful to then report back. To the patient. (Interview 003, pos. 68),
**Theme 3:** **Transferability of therapy settings into digital formats**	(93) you can generally use almost any intervention if you think about it properly—what is my goal with this intervention? Let’s put it this way, I wouldn’t think of anything in principle that can only be carried out in presence if you are open in the design. (Interview 004, pos. 60) (94) Mindfulness groups or something like that, that you teach them things for relaxation, that sort of thing. (Interview 002, pos. 52) (95) Things that you might also develop together, but also an overview to see what skills patients might fall back on if they are not feeling well (interview 007, pos. 46) (96) perhaps a nutrition log would be needed somehow to see what the daily calorie intake is, so to speak? I think that would also be very important (interview 007, pos. 46) (97) Somehow working on worksheets, resource work, in the direction of positive psychology, somehow strengthening self-efficacy or something, so that you can do something in that direction. (Interview 013, pos. 40) (98) perhaps also family discussions, these are already taking place here as part of studies (Interview 009, pos. 52) (99) I’m interested in a cooking course or something like that, I can definitely imagine digitally saying okay, today we’re cooking this and this dish together and then in a Zoom group event where the patients are accompanied and perhaps stand directly at the stove and prepare something. (Interview 016, pos. 44) (100) …Reduced app for cancer patients. That’s exactly why we developed these modules online… When they had completed one module, the next one was activated for them and so on, and there was always a survey at the beginning and a questionnaire at the end. (Interview 018, pos. 88)
**Subtheme 3.1:** **Individual therapy**	(101) …I can well imagine that you can also use video calls to do individual therapy … (Interview 007, pos. 52) (102) … individual therapy is common, and has already been integrated by various external providers or within the framework of other studies. (Interview 009, pos. 52)
**Subtheme 3.2:** **Group therapy**	(103) We have had very good experiences with group offers, although we also do it in such a way that we let the people on site come together in the group, because we have found that they are more open to each other once they have seen each other face to face. But that works really well. I think the people are relatively fit and confident thanks to the coronavirus period. … (Interview 008, pos. 58) (104) I perhaps believe that the inhibition threshold for people with social anxiety is a little lower to stay involved in group therapy. I can’t say whether it makes sense from a therapeutic point of view to do it like that or whether it wouldn’t make more sense to be more confrontational in person… But I could imagine that this might have a special appeal for them compared to face-to-face therapy. (Interview 001, pos. 62) (105) … I find that a bit difficult, because. I think it’s very easy to be very reserved in the group and not really use the group. In other words, you don’t use the group, so to speak, but rather just join in and that would be a bit more difficult to organize, especially when it comes to interactive groups, because there is simply less direct interaction. (Interview 015, pos. 95) (106) I believe that people come into better contact with each other there, because often when a group is on site, they see each other before it starts, get into conversation or something, and if it’s in the digital space, then they’re really only there for the minutes when the therapy is taking place and don’t have any private conversations. … And so in my experience, when groups were online like that, there was less flow of conversation. (Interview 018, pos. 74)
**Subtheme 3.3:** **Psychoeducation**	(107) I think psychoeducation is important … In other words, information events (interview 009, pos. 52) (108) psychoeducational elements to develop an understanding of the illness, to understand it. How does it work with my anxiety, with my trauma, with well, things like that, which are now also very doctor-led or nurse-led psychoeducational elements in relation to nutrition, so that the patient can also work out daily plans… (Interview 016, pos. 74) (109) I think that’s also an important element, exactly, and … modular offers, that you somehow, … have a module with various psychoeducational elements in it then another module with a small therapeutic task for the patients, which are then perhaps also specifically tailored to the individual. (Interview 023, pos. 66)
**Subtheme 3.4:** **Specialized therapy**	(110) I think what I find difficult are expressive things, artistic things. How do you want to make that available online? … everyone could somehow order their sound and make it and then discuss it somehow. But it’s a very isolated process and I can’t see what the other person is doing. So there’s less exchange possible or you always have to hold it up to the camera. …you’d have to look at sport and exercise … that’s also more difficult for me to implement and more through guided videos… (Interview 003, pos. 58) (111) In the case of special therapies, I see face-to-face teaching. So for me that is irreplaceable. It is necessary to accompany the patients so that they allow themselves to be touched, to be touched in the broadest sense, both emotionally and really, truly touched in this context. (Interview 010 D, pos. 84) (112) So let’s not kid ourselves, how engaging is it to watch a YouTube video with three yoga exercises or to sit in a group of twelve people and say we’re all going to do the downward facing dog? (Interview 003, pos. 62) (113) … When I see the lack of experience of movement, the lack of physical experience, the lack of understanding, there definitely needs to be an introduction in presence. … We’ve also decided against putting YouTube videos or something like that on there, because it simply requires guidance and support. I can’t imagine that in my area. (Interview 010, pos. 72) (114) Yes, we are now also planning to include exercise therapy. So far, we’ve only had an impulse lecture. I think it would be interesting if that were to be expanded a little, with some small exercises for everyday life, for example, that the movement therapist could do. …. So certainly also in music therapy. Art therapy can certainly be incorporated somehow. I don’t know how at the moment. (Interview 011 D, pos. 88)
**Subtheme 4.1:** **Bridging**	(115) So before there are no offers at all, yes, there is an absolute shortage of places. I could imagine that *it might be more time-efficient to bridge the gap.* I don’t see it as being replaceable yet (Interview 010, pos. 56) (116) that is to say, it would bridge this shortage of care. (Interview 013, pos. 34) (117) So it’s certainly a good interim solution. (Interview 020, pos. 52) (118) Especially for patients who somehow have long waiting times and need something acutely right now, I believe that many patient groups could benefit from this and I am fully in favor of offering therapy materials, individual sessions and also group sessions digitally, but not instead, but rather as an additional offer in the sense of bridging waiting times or doing something before patients would do nothing else. (Interview 013, pos. 76).
**Subtheme 4.2:** **Aftercare**	(119) That they still have a form of connection, that they can call again, because it happens quite often that they call … and that they can perhaps set up something like an aftercare program, that they can say I can contact them, that they can be seen again, that yes (Interview 002, pos. 94) (120) … afterwards in the interval they would have to go back for further treatment and then they could also come back again, as a day patient. You can then do that digitally, so that you can then say perhaps digitally on a day-care basis afterwards, they stay in their lives for a bit, but still get another intensive therapy that builds on that, that it’s complementary… (Interview 017, pos. 70)
**Subtheme 4.3:** **Hybrid approach**	(121) support therapy with digital media yes, replace no (Interview 15, pos. 119) (122) I actually don’t think so. I believe that this personal contact is an important factor and should remain so. But I also believe that you can’t generalize like that, you have to look at things individually. … But I don’t think that it would be completely replaced, at least not at the moment. (Interview 005, pos. 52) (123) … it can’t completely replace a ward, but it can complement it well. (Interview 011, pos. 48) (124) I could imagine it in the day clinic setting in particular. Individual content too. Doing it digitally or in the outpatient sector in particular. (Interview 015, pos. 51) (125) Which also include personal appointments, so that we can somehow get out of the digital realm a bit. But it could also be that you simply meet in person once a month and then make the rest of the appointments digitally or something like that. I think that can also be integrated a little bit. (Interview 017, pos. 74) (126) I believe that everyone would benefit if we were to combine the best of both world, replace certain things, and perhaps create more room for other things as a result. (Interview 021, pos. 60)
